# Acne Scar Subcision

**DOI:** 10.4103/0974-2077.69029

**Published:** 2010

**Authors:** BS Chandrashekar, AS Nandini

**Affiliations:** *Dr. Venkat Charmalaya, Centre for Advanced Dermatology, Bangalore, Karnataka, India*

**Keywords:** Subcision, acne scars, depressed scars

## Abstract

Subcision is a simple and safe office surgery procedure for treatment of depressed acne scars. It can easily be combined with other treatments such as laser, dermaroller and scar revisions for maximum efficacy.

## INTRODUCTION

Subcision, also called as subcutaneous incisionless surgery, a term coined by Orentreich and Orentreich in 1995[1] to describe the minor surgical procedure for treating depressed scars and wrinkles using a tri-beveled hypodermic needle inserted through a puncture in the skin surface (hence, “incisionless” surgery), and its sharp edges manoeuvred under the defect to make subcuticular cuts or “-cisions. The principle of this procedure is to break the fibrotic strands, which tether the scar to the underlying subcutaneous tissue. The depression is lifted by the releasing action of the procedure, as well as from connective tissue that forms during the course of normal wound healing.[1]

## INDICATIONS

It is mainly useful for rolling scars (distensible, depressed scars with gentle sloping edges).[2]

## PROCEDURE

**Figure 1:**
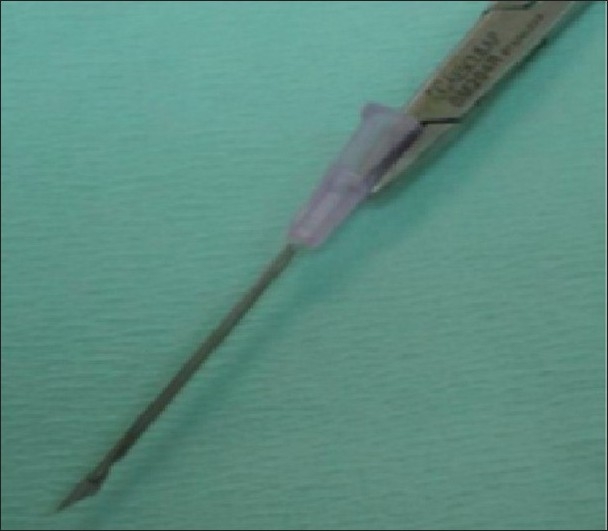
Nokor needle - Note the bevelled edge and its relation to the needle holder

Subcision is performed under local anaesthesia (topical or infiltration). Number 18 or 20 gauge needle or a Nokor needle (1.5 inch, 18-gauge, [Figure 1]) is inserted adjacent to the scar with the bevel upwards parallel to the skin surface, into the deep dermis and moved back and forth in a fan-like motion under the scar to release fibrous bands at dermal or deep dermal subcutaneous plane.[3–5] A snapping sound is heard as the fibrous bands are broken. Some authors recommend to and fro motion as in liposuction initially. The needle is removed and squeezed circumferentially around exit point to evacuate excess blood and prevent large haematoma formation. A small haematoma is allowed to be formed, which supports the released scar. Haemostasis is maintained with pressure and ice application.[3]

## POST-OPERATIVE CARE

Ice application on the operated site on the day, antibiotics and anti-inflammatory drugs for 5–7 days.[3]

## PRACTICAL TIPS

- The orientation of the needle during subcision should be horizontal. It often becomes necessary to withdraw the needle outside to be visible at the entry point and then to redirect it. There are different ways to ensure Nokor needle orientation:
  1. Holding the needle with a needle holder such that the horizontal orientation of the triangular tip of the Nokor needle is parallel to the horizontal plane of the needle holder gives constant control for the orientation without withdrawing it outside.[6]
  2. The hub of the needle is marked with 2 short straight lines, with a marker, perpendicular to the triangular cutting surface, at 12 and 6 o’clock position of the hub. If the orientation of the needle is lost due to rotation of the needle the operator needs only to adjust the hub so that the 2 marks are at a 12 and 6 o’clock position eliminating the need to withdraw the needle to visualise the tip. This technique can be used with or without a syringe attached to the hub of the Nokor needle.[7]
- Individual scars should be treated using separate multiple puncture sites.
- Care should be taken to avoid the preauricular, temporal and mandibular areas in order to avoid injuries to the facial nerve and major vessels.
- When multiple scars are undermined, the most dependent area is done first.
- Repeated sittings are required for better cosmetic results. Repeat sessions are done after 3-weeks interval.
- Patients should be counselled properly about haematoma and bluish discolouration
- If many scars need subcision, few scars may be treated at a time, like on one cheek to avoid severe oedema.
- Procedure is preferably done before a weekend or holiday for working patients.[3]

Subcision may be performed alone by itself or as an adjunct to other procedures like cryo slush, dermaroller, punch floatation, fractional pixel laser etc.[3] A study conducted to evaluate a novel subdermal filler “absorbable plain catgut suture” with subcision showed no additional benefits.[8] In a study of 22 patients, subcision was done on one side of the face and on the other side subcision with subdermal implant was done. The rate of response showed no significant difference with the use of subdermal implant.[8]

## SUMMARY

Subcision is a safe, simple technique that provides significant long-term improvement in the “rolling scars” of selected patients. It can be safely and easily combined with other treatments for acne scars.
